# Hæmorrhoidal Affections

**Published:** 1867-07

**Authors:** 


### *Hæmorrhoidal Affections.*

A. J. Baxter, M.D., a well-known correspondent of this journal, proposes, hereafter, to devote particular attention to this specialty. Without committing the JOURNAL to the advocacy of "specialties" we are glad to acknowledge Dr. B.'s skill and success in cases of the kind. See his advertisement.

